# Benefit or Harm? Arguments for and Against Early Detection of Dementia in Primary Care.

**DOI:** 10.1093/geroni/igaf122.1727

**Published:** 2025-12-31

**Authors:** Heather Schickedanz, Anna Chodos, Jarmin Yeh, Freddi Segal-Gidan

**Affiliations:** Harbor-UCLA Family Medicine, Harbor City, California, United States; University of California, San Francisco, San Francisco, California, United States; University of California, San Francisco, San Francisco, California, United States

## Abstract

There is an ongoing debate over the value of early dementia detection in primary care settings. Routine screening of adults aged 65+ aligns with the Affordable Care Act’s Annual Wellness Visit requirements but is in opposition to the 2020 U.S. Preventive Services Task Force’s “insufficient evidence”, and therefore negative, recommendation for universal screening. Proponents emphasize that over half of dementia cases go undiagnosed with people from racial and ethnic minority backgrounds disproportionately affected. They argue that primary care providers’ long-standing relationship with patients positions them well to identify subtle cognitive changes and implement early interventions, such as hearing correction, which may slow cognitive decline regardless of etiology. Early detection enables patients and families to better manage symptoms, access treatments or therapies, and engage in care planning for future needs. Opponents supports the U.S. Preventive Services Task Force’s position against routine screening, citing insufficient evidence of benefit. They argue that primary care providers face significant time constraints, and without clear post-diagnosis interventions, screening may be premature. They emphasize fundamental gaps in provider preparedness to identify cognitive impairment and provide support and resources. Opponents contend that widespread screening without robust dementia-capable infrastructure risks causing potential harm to patients and caregivers. This debate illuminates critical tensions between the potential benefits of early detection and healthcare system limitations. A full discussion, however, should extend beyond clinical considerations to encompass broader questions of health equity, workforce development, and system readiness. These complexities necessitate careful consideration of how to ensure the earliest actionable detection of dementia.

